# Suboptimal status of tummy time for infants in early childhood education institutions in urban China: A cross-sectional study

**DOI:** 10.7189/jogh.14.04048

**Published:** 2024-03-15

**Authors:** Yiwen Huang, Xiaotong Wang, Na Meng, Lin Li, Jian Zhang, Qiong Wu, Yanfeng Zhang

**Affiliations:** 1 Child Healthcare Centre, Children’s Hospital, Capital Institute of Paediatrics, Beijing, China; 2 Department of Integrated Early Childhood Development, Capital Institute of Paediatrics, Beijing, China; 3 Beijing KidsHome Children Development Centre, Beijing, China

## Abstract

**Background:**

Although tummy time is recommended as a form of physical activity for non-movable infants worldwide, little is known regarding the current status of tummy time practices among Chinese infants. Early childhood education (ECE) institutions provide children with rich learning experiences; however, tummy time practices among infants in these ECE institutions were unclear. This study aimed to investigate the status of tummy time among infants within the context of ECE institutions.

**Methods:**

We conducted a cross-sectional survey with primary caregivers of infants aged 0–11 months across 31 provinces of China from 1 March to 30 April 2023. To recruit participants, we collaborated with Gymboree Play & Music, an ECE institution with over 500 centres in nearly 200 cities in urban China. Our survey instrument was developed based on the World Health Organization (WHO) guidelines and literature to collect data on infants’ tummy time practices, caregivers’ tummy time knowledge and information sources. We used self-administered questionnaires through WeChat, in which participants scanned a quick response (QR) code to complete the questionnaire.

**Results:**

We included 1040 infants and their primary caregivers, with 504 infants aged 0–5 and 536 infants aged 6–11 months old. Less than half of infants (48.2%) started tummy time in the neonatal period, with 20.5% starting within two weeks after birth. Only 27.2% of infants engaged in at least 30 minutes of tummy time during the last 24 hours, with infants aged 0–5 months significantly lower than those aged 6–11 months (21.6 vs 32.5%, *P* < 0.0001). No significant difference was found between attending ECE class and non-attending ECE class groups for the proportion of infants with tummy time ≥30 minutes per day (28.9 vs 23.4%, *P* = 0.0625); however, infants aged 0–5 months in the attending group engaged in longer duration of tummy time than those in the non-attending group (*P* = 0.0005). The compliance with the tummy time guidelines in infants receiving long-nurturing care was significantly higher than those receiving short-nurturing care (30.4 vs 22.1%, *P* = 0.0036). Only 42.7% of caregivers knew that at least 30 minutes daily tummy time was necessary for infant, and more primary caregivers in the attending group knew that, compared to the non-attending group (45.3 vs 36.8%, *P* = 0.0098).

**Conclusions:**

The current status of infants’ tummy time practices and caregivers’ knowledge are generally suboptimal within the context of ECE institutions in urban China. Longer nurturing time contributes to higher compliance with tummy time guidelines. Effectively promoting tummy time practices through multiple channels in China is crucial.

The early development of healthy behaviours, such as engaging in physical activity, plays a vital role in preventing childhood overweight or obesity and promoting various aspects of well-being, including muscle strength, bone health, emotional well-being, and social cognition [1–3]. Furthermore, these behaviours have long-term effects on reducing the risk of chronic diseases in adulthood [1]. In 2019, the World Health Organization (WHO) published Guidelines on physical activity, sedentary behaviour and sleep for children under five years of age, recommending that ‘infants (less than one year old) should be physically active several times a day in a variety of ways, particular through interactive floor-based play; more is better. For those not yet mobile, this includes at least 30 minutes in the prone position (tummy time) spread throughout the day while awake.’ [4]. Evidence has shown that tummy time is positively associated with total development, gross motor development, and the ability to move while prone or supine (including crawling) [5]. However, in the traditional Chinese culture, sleeping flat heads in infants was popular, and Chinese caregivers tended to let their children spend too much time flat on the back rather than in prone position in early infancy, which may increase risk of positional plagiocephaly [6].

Recent studies in some Western countries indicated that the current status of tummy time practices remains suboptimal, with a low proportion of infants adhering to tummy time recommendations [7–10]. Studies conducted in UK and Australia found that half of parents started tummy time until four weeks after birth and no more than one-third of infants met the recommended 30 minutes of tummy time [7,8]. In addition, younger infants tend to exhibit a lower preference for engaging in tummy time activities, often displaying crying, rolling or squirming [9,11]. However, little is known regarding tummy time practices among infants in China and other Asian countries.

In 2021, China’s State Council released China Children’s Development Outline, stating that strengthening early childhood development service is a significant strategy to promote child health [12]. With the growing emphasis on early childhood development, early childhood education (ECE) institutions proliferated notably [13], which generally provide children with rich learning experiences to maximise motor, cognitive, and other development [14]. Primary caregivers opt for various types of ECE institutions to ensure comprehensive education and development for infants and young children, particularly in the urban areas of China [13]. Notably, in Shanghai, approximately 40% of children under three years old attended ECE institutions [15]. In certain Western countries, ECE institutions strictly adhere to guidelines regarding physical activities, and most institutions conform to these recommendations by incorporating dedicated tummy time sessions for infants [16]. However, ECE institutions in China demonstrate diverse class systems such as Montessori education, multiple intelligence theory, parent-teacher association, etc. [17], and tummy time practices among infants in these ECE institutions were unclear [18]. Therefore, our study aimed to investigate the knowledge and practices of tummy time among infants and their primary caregivers within the context of ECE institutions in urban China.

## METHODS

### Study design and participants

We conducted a cross-sectional survey across 31 provinces in China from 1 March to 30 April 2023 [19]. We used WeChat, version 8.0 (Tencent, Shenzhen, China) and self-administered questionnaires to collect information on infants’ tummy time practices and caregivers’ tummy time knowledge in urban China.

To recruit participants, we collaborated with Gymboree Play & Music, a prominent ECE institution that operates over 500 centres in approximately 200 urban cities throughout China [19]. Participants in this study were primary caregivers of infants 0–11 months old who were Gymboree Play & Music members attending ECE classes or non-members participating in classes and activities. We excluded caregivers who 1) did not have the WeChat application on their smartphones, 2) had infants older than 11 months, or 3) refused to participate in the survey.

### Sample size and sampling

We determined the sample size based on data from a previous study in the UK [7], assuming that 30% of infants would meet tummy time recommendations, with a desired level of absolute precision of 5%. Using a significance level of 5% and a power of 80%, the required sample size for the survey was calculated to be 676. Considering an estimated response loss of 30%, we aimed to recruit 966 participants by using convenience sampling method with the help of Gymboree Play & Music centre teachers. When membership caregivers took their children to attend ECE classes in the centres, or those caregivers who were non-members took their children to experience classes and activities, the teachers invited them to participate in the study.

### Survey instrument

Our survey instrument was developed based on the WHO recommendations, literature on tummy time [4,20] and expert consultation. With the questionnaire we assessed infants’ tummy time practices, caregivers’ tummy time knowledge, information sources on tummy time. In addition, we also collected information on infants’ activities during tummy time and caregivers’ attitudes when their infants were reluctant to keep a prone position. Moreover, we collected data on the frequency of infants attending ECE classes during the last month and primary caregivers’ daily nurturing care time (NCT). We pre-tested the survey instrument among 12 caregivers in December 2021. All the caregivers reported they were confused about the translation of tummy time. Therefore we added detailed explanation of tummy time in the questionnaire – tummy time is recommended to infants not yet mobile as a type of physical activity in prone position in which infants are awake and moving unstrained with the care of adults.

We set up the final questionnaire version on the Sojump platform, the largest professional online survey platform in China [21,22]. A quick response (QR) code linked to the questionnaire was also created for easy access.

### Data collection

**Figure 1:**
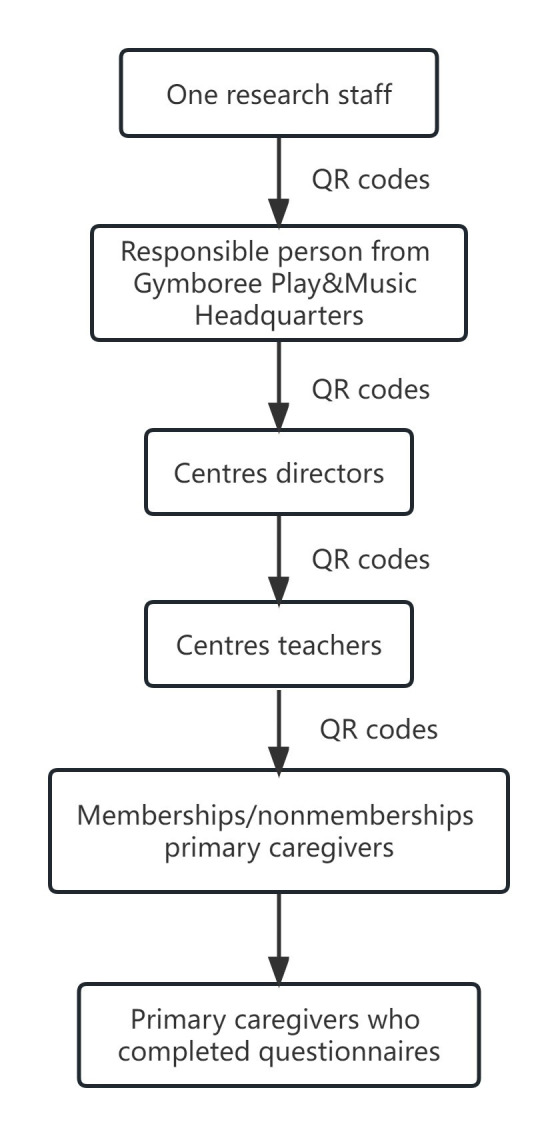
Flowchart of questionnaire dissemination.

We collected data with the assistance of Gymboree Play & Music centre teachers. First, a responsible person in Gymboree Play & Music headquarters received the QR code of questionnaire from our research team, and then distributed it to centre directors and subsequently to centre teachers. Teachers in each centre invited primary caregivers to participate in the survey (**Figure 1**). Once caregivers orally agreed to participate, teachers sent them the QR code, and caregivers scanned with their WeChat and completed the questionnaire online. The preceding paper presented a comprehensive and meticulous data collection process [23].

### Outcomes

The primary outcomes assessed were tummy time practices by two age groups (0–5 and 6–11 months old), including the duration and frequency of tummy time, and proportion of infants engaging in tummy time for at least 30 minutes during the last 24 hours and during last two weeks. Engaging in tummy time was defined as a prone position with or without activities. The literature reports that the infants’ average tummy time frequency was approximately 5.7 times at six months old [24]. We categorised the frequency of tummy time into three groups: no time spent, one to four times, and more or equal to five times.

The secondary outcome measured was the proportion of caregivers who knew about tummy time. Tummy time knowledge was evaluated based on responses to six questions about tummy time’s initiation, duration and benefits.

The third outcome was the differences in proportions of infants’ tummy time practice and caregivers’ tummy time knowledge between infants who attended ECE classes (attending group) and those who did not attend ECE classes (non-attending group) during the last month and the differences in proportions of infants’ tummy time practice and caregivers’ tummy time knowledge between infants whose primary caregivers’ daily nurturing care time ≥4 hours (long-NCT group) and those whose primary caregivers’ daily nurturing care time <4 hours (short-NCT group) during the last month. We also examined infants’ activities during tummy time, primary caregivers’ attitudes when faced with infants reluctant to assume a prone position, and sources of information on tummy time.

### Data management and statistical analysis

One research staff member (XW) managed the Sojump account database. The database in the Sojump account remained safe, and XW ensured that a unique password was maintained for the account. Upon completing and submitting questionnaires by primary caregivers, the answers were uploaded to the database in the Sojump account. After collecting the data, we downloaded the database and converted it into Microsoft Excel sheets for statistical analysis.

Data analysis was performed using SAS software, version 9.4 (SAS Institute, Cary, North Carolina, USA). We described categorical variables using numbers and proportions and continuous variables using medians and interquartile ranges. Differences in distribution were estimated using Wilcoxon rank sum test for continuous variables and χ^2^ test for categorical variables. The significance level was set at a *P*-value <0.05.

### Ethical approval

This study received approval from the Ethical Committee of the Capital Institute of Paediatrics in Beijing (SHERLL2022041). Before inviting participants to scan the QR code of the questionnaire, teachers in Gymboree Play & Music centres explained the study to caregivers. Upon scanning the QR code, primary caregivers were presented with an online informed consent form that required them to click on ‘agree to participate’ and provide an electronic signature before they answered the questions. Additionally, our team member secure Sojump account password and we signed a contract with Sojump company to secure the data and account.

## RESULTS

### Location and sample size

**Figure 2:**
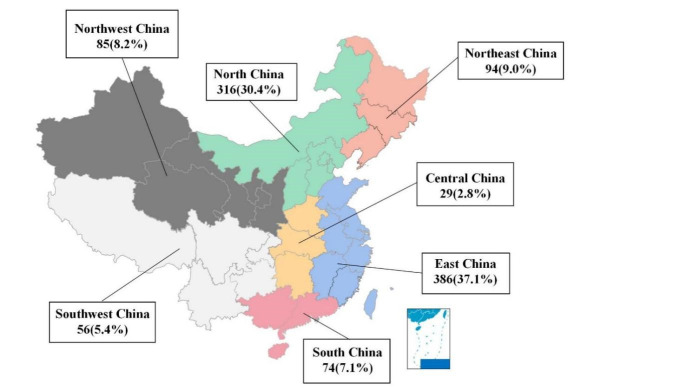
Geographical distribution of participants.

A total of 1040 primary caregivers and their infants participated in this survey, with 504 infants aged 0–5 months old and 536 infants aged 6–11 months old. The majority of primary caregivers came from East China (37.1%) and North China (30.4%), with the remaining one-third from the other five geographical areas **(****Figure 2**).

### Socio-demographic characteristics

Most infants were Han Chinese (92.1%) and male-to-female ratio was nearly equal. The attendance rate of ECE classes during the last month was higher among infants aged 6–11 months (77.8%) compared to those aged 0–5 months (59.9%). Most primary caregivers were mothers and fathers, accounting for 71.9% and 20.1%, respectively. Over 85% of primary caregivers attended college or above, and 61.7% spent more than four hours per day with infants during the last month (**Table 1**).

**Table 1:** Characteristics of surveyed infants and their primary caregivers*

	0–5 month old (n = 504)	6–11 month old (n = 536)	Total (n = 1040)
Infants			
**Sex**			
***Male***	**248 (49.2)**	**266 (49.6)**	**514 (49.4)**
***Female***	**256 (50.8)**	**270 (50.4)**	**526 (50.6)**
**Ethnicity**			
***Han***	**464 (92.1)**	**494 (92.2)**	**958 (92.1)**
***Ethnic minorities***	**40 (7.9)**	**42 (7.8)**	**82 (7.9)**
**Attended ECE classes during the last month**	**302 (59.9)**	**417 (77.8)**	**719 (69.1)**
**Primary caregivers**			
**Relationship**			
***Father***	**105 (20.8)**	**104 (19.4)**	**209 (20.1)**
***Mother***	**354 (70.2)**	**394 (73.5)**	**748 (71.9)**
***Grandparents***	**30 (6.0)**	**31 (5.8)**	**61 (5.9)**
***Other***	**15 (3.0)**	**7 (1.3)**	**22 (2.1)**
**Education**			
***Senior high school or below***	**67 (13.3)**	**88 (16.4)**	**155 (14.9)**
***College or above***	**437 (86.7)**	**448 (83.6)**	**885 (85.1)**
**Primary caregivers’ daily nurturing care time during the last month**			
***≥4 h/d***	**324 (64.3)**	**318 (59.3)**	**642 (61.7)**
***<4 h/d***	**180 (35.7)**	**218 (40.7)**	**398 (38.3)**


ECE – early childhood education

*Presented as n (%) unless specified otherwise.

### Practices and knowledge of tummy time

Regarding tummy time practices, approximately half of infants (48.2%) started tummy time during the neonatal period, with 20.5% starting early within two weeks after birth. The median duration of tummy time was 10 minutes for infants aged 0–5 months and 12 minutes for infants aged 6–11 months during the last 24 hours, demonstrating a significant difference between these age groups (*P* < 0.0001). Only 27.2% of total infants engaged in at least 30 minutes of tummy time during the last 24 hours, with a lower proportion of infants aged 0–5 months than those aged 6–11 months (21.6 vs 32.5%, *P* < 0.0001). Most infants (69.0%) practised tummy time one to four times during the last 24 hours, and frequencies of tummy time (more or equal to five times) in infants of 6–11 months were higher than in infants of 0–5 months (28.6 vs 21.2%, *P* = 0.0065). Tummy time practice indicators during the last two weeks showed similar patterns. For tummy time knowledge, 79.2% of primary caregivers acknowledged the importance of tummy time for infants’ development, but only 42.7% caregivers knew that at least 30-minute’ daily tummy time was necessary for infants. Proportion of primary caregivers knowing other four questions ranged from 48.7–69.6%. No differences in tummy time knowledge were observed between the two age groups (**Table 2**).

**Table 2:** Tummy time practices and knowledge of surveyed infants and their primary caregivers*

Tummy time practices and knowledge	0–5 month old (n = 504)	6–11 month old (n = 536)	Total (n = 1040)	*P*-value	
Initiation of tummy time				0.0236
*<2 weeks*	107 (21.2)	106 (19.8)	213 (20.5)	
*2–4weeks*	134 (26.6)	154 (28.7)	288 (27.7)	
*4–8weeks*	116 (23.0)	86 (16.1)	202 (19.4)	
*≥8 weeks*	66 (13.1)	95 (17.7)	161 (15.5)	
*Unknown*	69 (13.7)	87 (16.2)	156 (15.0)	
*Not yet started*	12 (2.4)	8 (1.5)	20 (1.9)	
Tummy time during the last 24 h				
*Duration of tummy time in minutes; MD (IQR)*	10 (5–20)	12 (5–30)	10 (5–30)	<0.0001
*The proportion of infants with tummy time ≥30 min*	109 (21.6)	174 (32.5)	283 (27.2)	<0.0001
Frequencies of tummy time				0.0035
*0*	44 (8.7)	33 (6.2)	77 (7.4)	
*1–4*	363 (72.0)	355 (66.2)	718 (69.0)	
*≥5*	97 (19.3)	148 (27.6)	245 (23.6)	
Tummy time during the last two weeks				
*Duration of daily tummy time in minutes; MD (IQR)*	10 (5–20)	15 (5–30)	10 (5–30)	<0.0001
*The proportion of infants with tummy time ≥30 min/d*	115 (22.8)	190 (35.4)	305 (29.3)	<0.0001
Frequencies of tummy time per day				0.0133
*0*	47 (9.3)	36 (6.7)	83 (8.0)	
*1 ~ 4*	350 (69.5)	347 (64.7)	697 (67.0)	
*≥5*	107 (21.2)	153 (28.6)	153 (25.0)	
Tummy time knowledge				
*Babies can be put on their tummies the day they are brought home from the hospital.*	310 (61.5)	356 (66.4)	666 (64.0)	0.0991
*I am confident that my baby is safe when I place him/her on his/her tummy while they are awake.*	242 (48.0)	264 (49.3)	506 (48.7)	0.6898
*More than 5 min a day on his/her tummy could not cause a baby harm.*	251 (49.8)	292 (54.5)	543 (52.2)	0.1314
*Tummy time should be more than 30 min/d.*	202 (40.1)	242 (45.1)	444 (42.7)	0.0985
*Tummy time when babies are awake is important for their development.*	402 (79.8)	422 (78.7)	824 (79.2)	0.6822
*Tummy time can prevent platycephaly and plagiocephaly.*	352 (69.8)	372 (69.4)	724 (69.6)	0.8779

IQR – interquartile range, MD – median

*Presented as n (%) unless specified otherwise.

For tummy time practices, the duration of tummy time in the attending group was significantly higher than in the non-attending group among infants aged 0–5 months during the last 24 hours and during the last two weeks. No significant difference was found between attending and non-attending groups for the proportion of infants with tummy time ≥30 minutes per day. Proportion of infants with tummy time ≥5 times per day in attending group was generally lower than in non-attending group, however, the significant difference was only found in infants aged 6–11 months (25.4 vs 35.3%, *P* = 0.0336). For tummy time knowledge, more primary caregivers in the attending group knew that tummy time should be more than 30 minutes per day compared to the non-attending group among infants aged 0–5 months (45.0 vs 32.7%, *P* = 0.0055) and in the total group (45.3 vs 36.8%, *P* = 0.0098). No significant differences between the two groups were found for other indicators of tummy time knowledge (**Table 3**).

**Table 3:** Comparison of tummy time practices and knowledge between infants who attended (attending group) and not attended ECE classes (non-attending group)*

	0–5 month old			6–11 month old			Total		
	**Attending group (n = 302)**	**Non-attending group (n = 202)**	***P*-value**	**Attending group (n = 417)**	**Non-attending group (n = 119)**	***P*-value**	**Attending group (n = 719)**	**Non-attending group (n = 321)**	***P*-value**
**Tummy time practices**									
Tummy time during the last 24 h									
*Duration of tummy time in minutes; MD (IQR)*	10 (5–25)	7 (2–20)	0.0005	10 (5–30)	15 (3–30)	0.6727	10 (5–30)	10 (3–25)	0.0005
*The proportion of infants with tummy time ≥30 min/d*	71 (23.5)	38 (18.8)	0.2093	137 (32.9)	37 (31.1)	0.7174	208 (28.9)	75 (23.4)	0.0625
*Frequencies of tummy time (≥5 times per day)*	58 (19.2)	39 (19.3)	0.9774	106 (25.4)	42 (35.3)	0.0336	164 (22.8)	81 (25.2)	0.3948
Tummy time during the last two weeks									
*Tummy time duration in minutes; MD (IQR)*	10 (5–30)	8 (2–20)	0.0016	15 (5–30)	15 (4–30)	0.8878	10 (5–30)	10 (3–30)	0.0012
*The proportion of infants with tummy time ≥30 min/d*	76 (25.2)	39 (19.3)	0.1246	144 (34.5)	46 (38.7)	0.4069	220 (30.6)	85 (26.5)	0.1778
*Frequencies of tummy time (≥5 times per day)*	62 (20.5)	45 (22.3)	0.6383	111 (26.6)	42 (35.3)	0.0646	173 (24.1)	87 (27.1)	0.2954
Tummy time knowledge									
*Babies can be put on their tummies the day they are brought home from the hospital.*	190 (62.9)	120 (59.4)	0.4277	279 (66.9)	77 (64.7)	0.6539	469 (65.2)	197 (61.4)	0.2310
*I am confident that my baby is safe when I place him/her on his/her tummy while they are awake.*	144 (47.7)	98 (48.5)	0.8545	206 (49.4)	58 (48.7)	0.8988	350 (48.7)	156 (48.6)	0.9808
*More than 5 min a day on his/her tummy could not cause a baby harm.*	152 (50.3)	99 (49.0)	0.7713	222 (53.2)	70 (58.8)	0.2804	374 (52.0)	169 (52.7)	0.8507
*Tummy time should be more than 30 min/d.*	136 (45.0)	66 (32.7)	0.0055	190 (45.6)	52 (43.7)	0.7182	326 (45.3)	118 (36.8)	0.0098
*Tummy time when babies are awake is important for their development.*	243 (80.5)	159 (78.7)	0.6317	326 (78.2)	96 (80.7)	0.5575	569 (79.1)	255 (79.4)	0.9118
*Tummy time can prevent platycephaly and plagiocephaly.*	214 (70.9)	138 (68.3)	0.5420	284 (68.11)	88 (74.0)	0.2224	498 (69.3)	226 (70.3)	0.7114

IQR – interquartile range, MD – median

*Presented as n (%) unless specified otherwise.

For tummy time practices, all three indicators during the last 24 hours in long-NCT group were significantly higher than those in short-NCT group in infants aged 6–11 months, and in total infants. Similar patterns were shown during the last two weeks. However, in infants aged 0–5 months, only tummy time duration and frequencies ≥5 times per day (23.2 vs 12.2%, *P* = 0.0029) during the last 24 hours in the long-NCT group were higher than in the short-NCT group. For tummy time knowledge, the proportion of primary caregivers who knew about tummy time was significantly lower in the short-NCT group in all age groups except for question four (**Table 4**).

**Table 4:** Comparison of tummy time practices and knowledge between infants with primary caregivers’ nurturing care time ≥4 h (long-NCT group) and primary caregiver’s nurturing care time <4 h (short-NCT group)*

	0–5 month old			6–11 month old			Total		
**Tummy time practices and knowledge**	**Long-NCT group (n = 324)**	**Short-NCT group (n = 180)**	***P*-value**	**Long-NCT group (n = 318)**	**Short-NCT group (n = 218)**	***P*-value**	**Long-NCT group (n = 642)**	**Short-NCT group (n = 398)**	***P*-value**
Tummy time practices									
*During the last 24 h*									
*Duration of tummy time in minutes; MD (IQR)*	10 (5–25)	8.5 (3–20)	0.0163	15 (5–30)	10 (5–30)	0.0372	10 (5–30)	10 (5–20)	0.0042
*The proportion of infants with tummy time ≥30 min/d*	77 (23.8)	32 (17.8)	0.1177	118 (37.1)	56 (25.7)	0.0055	195 (30.4)	88 (22.1)	0.0036
*Frequencies of tummy time (≥5 times per day)*	75 (23.2)	22 (12.2)	0.0029	105 (33.0)	43 (19.7)	0.0007	180 (28.0)	65 (16.3)	<0.0001
During the last two weeks									
*Tummy time duration in minutes; MD (IQR)*	10 (5–22)	10 (3–20)	0.1019	20 (5–30)	10 (5–30)	0.0255	10 (5–30)	10 (5–25)	0.0133
*The proportion of infants with tummy time ≥30 min/d*	78 (24.1)	37 (20.6)	0.3671	128 (40.3)	62 (28.4)	0.0050	206 (32.1)	99 (24.9)	0.0130
*Frequencies of tummy time (≥5 times per day)*	77 (23.8)	30 (16.7)	0.0619	109 (34.3)	44 (20.2)	0.0004	186 (29.0)	74 (18.6)	0.0002
Tummy time knowledge									
*Babies can be put on their tummies the day they are brought home from the hospital.*	216 (66.7)	94 (52.2)	0.0014	224 (70.4)	132 (60.6)	0.0172	440 (68.5)	226 (56.8)	0.0001
*I am confident that my baby is safe when I place him/her on his/her tummy while they are awake.*	170 (52.5)	72 (40.0)	0.0073	186 (58.5)	78 (35.8)	<0.0001	356 (55.5)	150 (37.7)	<0.0001
*More than 5 min a day on his/her tummy could not cause a baby harm.*	181 (55.9)	70 (38.9)	0.0003	193 (60.7)	99 (45.4)	0.0005	374 (58.3)	169 (42.5)	<0.0001
*Tummy time should be more than 30 min/d.*	132 (40.7)	70 (38.9)	0.6844	140 (44.0)	102 (46.8)	0.5276	272 (42.4)	172 (43.2)	0.7880
*Tummy time when babies are awake is important for their development.*	274 (84.6)	128 (71.1)	0.0003	265 (83.3)	157 (72.0)	0.0017	539 (84.0)	285 (71.6)	<0.0001
*Tummy time can prevent platycephaly and plagiocephaly.*	239 (73.8)	113 (62.8)	0.0100	232 (73.0)	140 (64.2)	0.0311	471 (73.4)	253 (63.6)	0.0008

IQR – interquartile range, MD – median, NCT – nurturing care time

*Presented as n (%) unless specified otherwise.

**Figure 3:**
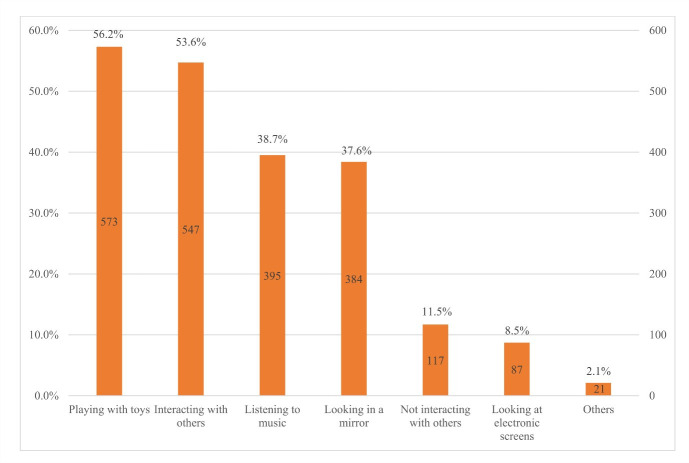
Activities of infants during tummy time.

**Figure 4:**
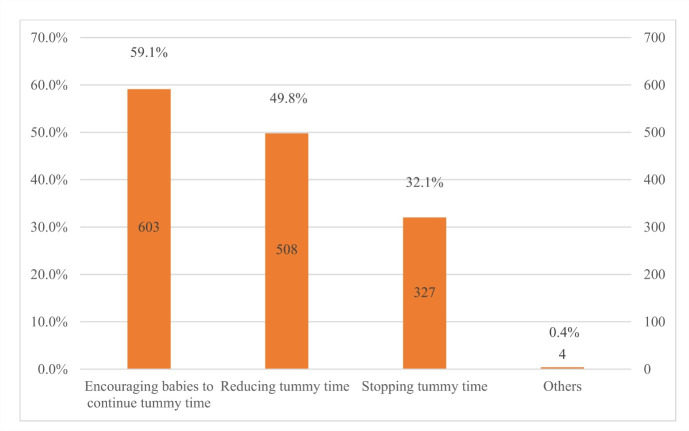
Primary caregivers’ response when infants are reluctant to lie on their tummies.

**Figure 5:**
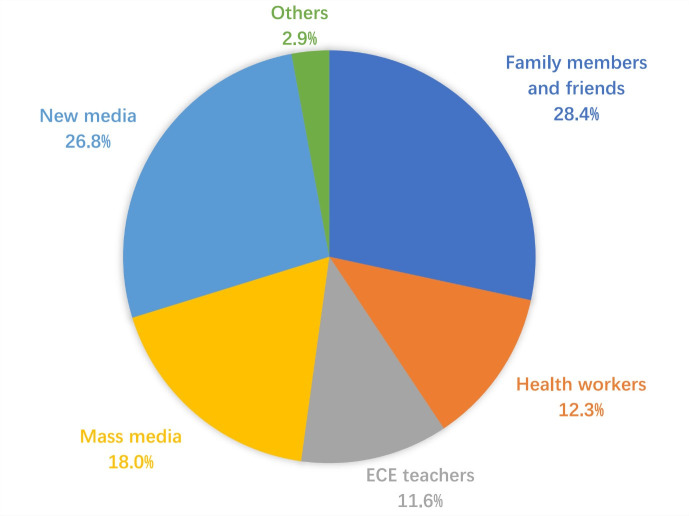
Source of information on tummy time.

More than half of infants were playing with toys (56.2%) during tummy time, followed by interacting with other people (53.6%), listening to music (38.7%) and looking in a mirror (37.6%). However, 11.5% of infants did not interact with others, and 8.5% looked at electronic screens. Primary caregivers’ responses varied when infants were reluctant to lie on their tummies. Nearly 60% of primary caregivers encouraged their babies to continue tummy time, whereas half reduced it. Only 32.1% of primary caregivers stopped tummy time immediately. Primary caregivers mainly received information on tummy time from family members and friends (28.4%), followed by news media (26.8%), mass media (18.0%), health workers (12.3%) and ECE teachers (11.6%) (**Figure 3**, **Figure 4**, and **Figure 5**).

## DISCUSSION

This study presents the first comprehensive investigation of tummy time practices, knowledge, and information sources among Chinese infants and their primary caregivers. Our findings indicate that the duration, frequency, and initiation of tummy time are generally suboptimal in this population. No significant differences were found among infants whether attending ECE classes. However, compared to infants receiving less than four hours of daily nurturing care, those with longer nurturing care time exhibited higher compliance with tummy time guidelines.

Our findings indicate that less than one-third of infants meet the WHO recommendation of a minimum daily tummy time of 30 minutes, and approximately 10% of infants engaged in unhealthy activities such as screen time during their tummy time sessions. These findings are consistent with recent studies conducted in countries that have proposed guidelines for children’s movement behaviour, which indicated that 31% of infants in the UK and 29.7% of infants in Australia achieved the recommended daily duration of tummy time [7,10]. Further, in our study, half of the primary caregivers were unaware of the recommended 30 minutes of tummy time per day and expressed concerns about its potential harm. This lack of awareness and concern might significantly contribute to low adherence to tummy time. A previous study also revealed that some parents refrain from placing infants in the prone position due to their intolerance, and educating parents about the significance and early initiation of tummy time represents a more effective approach for reducing the risk of intolerance [25].

To meet the demands of family education and nurture the potential of young infants, an increasing number of Chinese parents are choosing to enrol their infants in ECE institutions [13]. However, no significant differences were observed in tummy time practices and knowledge among infants whether attending these institutions, which contradicts our findings that young children who participated in ECE classes exhibited significantly greater adherence to recommendations on duration of physical activity and outdoor activity [23]. Furthermore, only 11.6% of primary caregivers identified ECE teachers as a source of information on tummy time, indicating a lack of awareness among teachers in Chinese ECE institutions regarding WHO recommendations. On the contrary, policies mandating daily tummy time for infants have been published by 16 states in the USA [16]. A compliance survey conducted across childcare centres in Australia, USA, and Canada revealed that 74–95% of institutions implemented tummy time sessions [16,26]. These findings suggest that Chinese ECE institutions should incorporate sufficient tummy time into their curriculum, promote home-based practice methods, and emphasise infant developmental benefits.

Nurturing care provided by parents and family interactions in the early years establishes a foundation for lifelong health, growth, and development while reducing the risk of injury [27,28]. We observed that infants who received longer periods of nurturing care from their primary caregivers were more actively engaged in tummy time than those with shorter periods. This finding aligns with our previous results that infants who experience extended nurturing care demonstrate better adherence to physical activity guidelines [23]. As a stable environment, nurturing care offers opportunities for early learning, emotional support and developmental interactions [27,28]. Therefore, infants in such an environment will likely have increased access to physical activities and interactive games.

We observed that 70% of infants attended ECE classes in the past month. Moreover, young infants and their caregivers in China can access health facilities for regular prenatal and postnatal check-ups [19]. However, ECE teachers and health workers accounted for less than a quarter of all information sources related to tummy time. Given the high-frequency contact with ECE teachers and health workers, we recommend enhancing ECE institutions and health facilities as delivery channels of tummy time information by involving ECE teachers in teaching home-based practices and having health workers provide knowledge during clinic visits. In contrast to ECE teachers and health workers, new media, mass media, and interpersonal sources (such as relatives and friends) were identified in our study as the primary channels through which individuals seek health information. This observation aligns with a systematic review previously described [29]. As people increasingly rely on social media platforms, more individuals consult various sources of information to address their health concerns, read about others’ experiences, and access relevant health resources online [29]. However, ensuring the reliability and quality of online information is crucial due to its potential detrimental effects [30].

This study represents the first investigation in urban China, providing fundamental insights into tummy time practices within the country. However, our study has several limitations. The questionnaire survey relies on caregivers’ recall during the last 24 hours and two weeks, which may lead to recall bias regarding the accuracy of tummy time duration compared to objective accelerometers [11,24]. Further, we utilised convenience sampling rather than randomised sampling and solely collected data through ECE institutions, which may lead to biases in results; therefore, caution should be exercised when generalising the findings [31]. However, such a large-scale and broad-covered survey fills the gap of tummy time practices in China, and provides evidence for future intervention strategy aimed at improving tummy time practices and health outcomes for infants.

## CONCLUSIONS

Our study indicates that infants’ tummy time practices and caregivers’ tummy time knowledge are generally suboptimal in ECE institutions in urban China. Longer nurturing time contributes to higher compliance with tummy time guidelines. It is crucial to promote tummy time practices through multiple channels effectively in China.
